# Prevalence and Risk Factors of Osteoporosis among Postmenopausal Women Visiting a District Hospital of Nepal: An Observational Study

**DOI:** 10.31729/jnma.8800

**Published:** 2024-11-30

**Authors:** Dipendra Pandey, Samina Basnet, Smeena Pradhananga, Sarita Shrestha, Badri Rijal, Aman Neupane, Utsav Timilsina, Amit Upreti, Apil Upreti, Rabindra Baskota, Pawan Kumar Hamal

**Affiliations:** 1 Department of Orthopaedics, Trishuli Hospital, Trishuli, Nuwakot, Nepal; 2 Trishuli Hospital, Trishuli, Nuwakot, Nepal; 3 Department of Obstetrics and Gynecology, Trishuli Hospital, Trishuli, Nuwakot, Nepal; 4 Department of Internal Medicine, Trishuli Hospital, Trishuli, Nuwakot, Nepal; 5 Department of Orthopedics, National Trauma Center, Mahankal, Kathmandu, Nepal; 6 Trishuli Hospital, Trishuli, Nuwakot, Nepal; 7 Special School for Disabled and Rehabilitation Center, Old Sinamangal, Kathmandu, Nepal; 8 National Neuro Center, Maharajgunj, Kathmandu, Nepal; 9 Department of Health Services, Teku, Kathmandu, Nepal; 10 Department of Anaesthesiology, Trishuli Hospital, Trishuli, Nuwakot, Nepal

**Keywords:** *bone mineral density*, *osteoporosis*, *prevalence*, *risk factors*

## Abstract

**Introduction:**

Osteoporosis and low bone mass affect millions of people worldwide, leading to severe consequences ranging from disability to mortality. This study aimed to determine the prevalence and risk factors of osteoporosis among postmenopausal women in a district of Nepal.

**Methods:**

An analytical cross-sectional study involving postmenopausal women from Nuwakot, Dhading, and Rasuwa districts in Nepal was conducted at Trishuli Hospital, Nuwakot. Ethical approval was taken from the Institutional Review Board of Nepal Health Research Council (Reference number: 1768). The prevalence of osteoporosis was determined, and the associated factors were analyzed using multivariate logistic regression. Dual-Energy X-ray Absorptiometry (GE-Lunar Prodigy) was used to measure Bone Mineral Density (g/cm^2^) at the proximal femur and lumbar spine. Various factors related to osteoporosis were also analyzed.

**Results:**

There were 384 postmenopausal women and the prevalence of osteoporosis was 82 (21.35%; 95% CI: 17.25%-25.45%)e. The mean age of female with osteoporosis was 67.52±8.84 years and that without osteoporosis was 55.70±7.69 years (p <0.001). The multivariate logistic regression showed aOR 0.82 for body mass index.

**Conclusions:**

The study reports a lower prevalence of osteoporosis than expected in postmenopausal women. There was a significant inverse relationship between osteoporosis and body mass index. However, no significant association was observed between Bone Mineral Density, biochemical variables, smoking, and parity.

## INTRODUCTION

Osteoporosis is a metabolic disease characterized by low bone mass, structural deterioration of bone tissue and compromised bone strength. It affects millions of people worldwide.^1^ Factors contributing to osteoporosis include dietary calcium intake, vitamin D deficiency, reduced physical activity, low Body Mass Index (BMI), and deranged thyroid function.^2-9^ The prevalence of osteoporosis increases with age, particularly among postmenopausal women because of their hypo-estrogenic state.^10^

Studies show that 10 million people over the age of 50 have osteoporosis in the USA.^11^ Prevalence of osteoporosis in elderly women was reported as 15% in Germany, 38% in Japan, 8% in France, and 9.65% in China.^12^ Recent studies also show a rising trend in the prevalence of the disease in Southeast Asian countries including Nepal.^13,14^

This study aims to add to existing database by finding prevalence of osteoporosis in postmenopausal women in rural Nepal using DEXA scans and examine its association with biochemical parameters such as Vitamin D, serum Calcium, and serum Albumin.

## METHODS

This analytical cross-sectional study included postmenopausal women from three districts (Nuwakot, Rasuwa, and Dhading) of Nepal, who were invited for voluntary participation. Participants of all municipalities of the three districts, based on their proximity to Trishuli Hospital, which served as the primary site for conducting DEXA scans and blood tests., were chosen. These municipalities were chosen to ensure accessibility for participants and to capture a representative sample of postmenopausal women from rural areas, while also considering logistical and resource constraints. The study was initiated after ethical approval from the Ethical Review Board of Nepal Health Research Council (Reference number: 1768). Postmenopausal women were defined as those experiencing at least 1 year of menopause. Ambulatory women who provided written consent to the study as well as to providing blood samples were included in the study, while those who had undergone hormone replacement therapy or post-hysterectomy, and nonambulatory participants who couldn't voluntarily come to the hospital were excluded. Convenience sampling was conducted by selecting participants who were easily accessible to the researchers. The target population for this study comprised all eligible postmenopausal women from Nuwakot, Dhading, and Rasuwa districts, who were invited to participate. However, the total population analyzed in the study included only those women who presented to Trishuli Hospital and underwent the DEXA scan.

To include the maximum sample size 50% prevalence of osteoporosis was considered and sample size was calculated by using the following formula.


$$\begin{array}{ll} \text{n} & ={\text{Z}}^{2}\times \frac{\text{p}\times \text{q}}{{\text{e}}^{\text{2}}}\\ & =1.96^{2}\times \frac{\text{0.50}\times \text{0.50}}{0.05^{2}}\\ & =384 \end{array}$$

Where,

Z = 1.96 at 95% Confidence Interval (CI)
p = 50% for maximum sample size calculation
q = 1-p
e = 5% margin of error

Therefore the obtained sample size was 384. For the multivariate logistic regression, an effect size (f^2^) of 0.15, an alpha of 0.05, a power of 0.95, and 4 predictors were considered. The G*Power analysis recommended a minimum sample size of 129, which supports adequacy of our sample for this model.

Postmenopausal women were selected from the study districts by disseminating information through local radio stations and social media. Furthermore, local government authorities, health coordinators, and Female Community Health Volunteers (FCHV) were contacted and requested to spread the information and gather the participants at Trishuli Hospital, Nuwakot where the DEXA scan and blood tests were done. The study was conducted from March 2023 to June 2023.

Data was collected using a questionnaire, which included information on age, age at menarche, age at menopause, duration of menopause, parity, current smoking habits, weight, and height. Weight and height were measured using a digital weighing machine and vertical scale, respectively. BMI was calculated as body weight (kg)/height (m^2^) and classified as underweight (<18.5 kg/m^2^), healthy weight (18.5 to 24.9kg/m^2^), overweight (25 - 29.9 kg/m^2^), and obesity (>/= 30 kg/m^2^).^15^

Bone mineral density (BMD) was assessed using the Dual-energy X-ray absorptiometry (DEXA) technique with the GE Lunar Prodigy machine. The measurements were taken at the neck of the femur and the lumbar spine. Trained technicians conducted all the examinations, ensuring accuracy and precision, and they performed daily calibrations of the densitometers using equipment-specific phantoms.

To define osteoporosis, the criteria set forth by the World Health Organization (WHO) were adopted for this study. Osteoporosis was identified when the BMD T-score was found to be less than or equal to -2.5, as determined by DEXA. On the other hand, osteopenia, a milder form of bone loss, was characterized by a bone density falling between -1 and -2.5 standard deviations below the reference point.^16^

Blood samples were collected from all the participants. Qualified laboratory technicians then conducted measurements of serum calcium, serum albumin, and Vitamin D levels using established and standardized methods.

Statistical analyses were performed using IBM SPSS. Descriptive statistics, including mean and standard deviations, were calculated for continuous variables and 95% Confidence Interval (CI) was calculated. The variables found to be significant in bivariate analysis were considered for multivariable analysis. Multiple logistic regression analysis was used to identify factors predicting osteoporosis, with a probability value of <0.25 accepted as the level of statistical significance.

## RESULTS

The study included 384 participants, among whom 82 (21.35%; 95% CI: 17.25%-25.45%) were found to have osteoporosis. Among them, 95 (24.74%) had normal bone mineral density, and 207 (53.91%) were osteopenic (Table 1).

The mean age of the postmenopausal women with osteoporosis is 67.52 ±8.84 years with the mean age of menarche and menopause being 15.71±2.15 and 46.91±6.53 years, respectively. The mean BMI and BMD were 24.01±4.58 kg/ m^2^ and 0.59±0.09 g/cm^2^, respectively (Table 2).

**Table 1:** Prevalence of osteoporosis and osteopenia among postmenopausal women (n = 384).

Variable	n (%)
Normal	95 (24.74)
Osteopenia	207 (53.91)
Osteoporosis	82 (21.35)

**Table 2:** Demographic and clinical characteristics of postmenopausal women (n = 384).

Variable	Women with osteoporosis (n = 82)	Women without osteoporosis (n = 302)	p value
Current age^*^	67.52±8.84	55.70±7.69	<0.001
Age at Menarche	15.71±2.15	15.19±1.92	0.791
Age at Menopause	46.91±6.53	47.12±5.35	0.37
Parity^*^	4 (3-6)	3 (2-4)	0.01
BMI (kg/m2)^*^	24.01±4.58	27.06±4.28	<0.001
BMD (g/cm2)^~^	0.59±0.09	0.86±0.11	<0.001
Smoking^*^	22 (26.83)	57	0.114
Albumin (gm/dl)	2.77±0.86	2.80±0.48	0.776
Calcium (mg/dl)	8.51±1.03	8.43±1.01	0.521
Vitamin D (ng/mL)	27.39±5.39	27.41±3.81	0.982

* <0.25 is taken as significant

~ For diagnosis of osteoporosis, BMI = Body Mass Index, BMD = Bone Mineral Density

**Table 3:** Multivariate logistic regression analysis of variables.

Variables	aOR	p value	Confidence Interval
Age^*^	1.19	<0.001	1.14-1.24
Parity	0.98	0.803	0.85-1.14
Smoking	1.52	0.248	0.75-3.09
BMI^*^	0.82	<0.001	0.75-0.89

* Statistical significance at p<0.05, BMI = Body Mass Index, aOR = Adjusted ODDS Ratio

Among the four variables analyzed age demonstrated statistically significant association with osteoporosis and BMI showed a statistically significant inverse association with osteoporosis (p<0.001) with an aOR of 0.82 (95% CI: 0.750.89). However, parity and smoking show lack of statistical significance imply uncertainty in this association (Table 3).

## DISCUSSION

The study revealed that 82 (21.35%; 95% CI:17.25%-35.45%)of postmenopausal women had osteoporosis. The finding is similar to the study conducted among the women of central Nepal where BMD was measured using Mini Omni quantitative ultrasound technology, that reported the prevalence of osteopenia and osteoporosis at 39.3% and 26.2%, respectively.^12^ The sample size of 384 in this study allowed for a meaningful statistical analysis of the postmenopausal women from the three districts. The study's inclusion criteria that defined postmenopausal women as those who had experienced at least one year of menopause, was also vital in ensuring that the sample accurately reflected the individuals who were at a higher risk of osteoporosis. However, while the convenience sampling method allowed for accessibility and participation from various local levels, it might have introduced bias regarding representativeness.

The findings of this cross-sectional study provide valuable insights into the prevalence of osteoporosis among postmenopausal women in the Nuwakot, Rasuwa, and Dhading districts of Nepal. Osteoporosis is a significant health issue, especially among postmenopausal women, as hormonal changes during menopause have been linked to decreased bone mineral density.^6,12^ Understanding the factors influencing BMD in this population is crucial for early detection, prevention, and management of osteoporosis-related complications.

In this study, the mean age of the postemenopausal women with osteoporosis was found to be 67.52±8.84 years and increasing age was found to have significant positive correlation with higher osteoporosis risk (AOR<0.001), supporting previous research indicating an age-related decline in BMD in women.^4,6,13^ Advancing age is a well-established risk factor for osteoporosis, and the study's findings reinforce the need for targeted interventions in older women to maintain bone health and reduce fracture risk.^9^

The study also highlighted the statically significant inverse relationship between BMI and BMD. Women with higher BMI were less likely to have osteoporosis, suggesting a potential protective effect of increased body weight on BMD. These findings align with existing evidence that have discussed this protective role existing due to mechanical loading of bones.^4^ This association demonstrates the importance of maintaining a healthy BMI to support bone health.

Additionally, while some previous studies have suggested that a higher parity results in low BMD in the lumbar spine, parity, while showing some association, was not statistically significant in the multivariate analysis conducted for this study.^17^ From this discrepancy, it can be inferred that further studies involving diverse populations are needed to understand the effect of reproductive history on BMD. Similarly, menstrual history–age at menarche and menopause–showed no significant difference between the groups with or without osteoporosis. However, age at menopause has been linked to a higher risk of bone loss in postmenopausal women and a low bone density has been linked to late menarche in premenopausal women but not in the postmenopausal population in different studies.^7,18^ These results highlight the importance of hormonal changes during menopause as key factors in BMD decline. Healthcare providers should consider menopausal history in risk assessment and preventive strategies.

Despite conducting various analyses of various biochemical parameters in this study, none demonstrated significant associations with BMD, which is consistent with a similar study conducted in China.^19^ In contrast, there are evidence suggesting that low serum calcium can lead to osteoporosis resulting from increased bone reabsorption.^6,20^ Similarly, low vitamin D levels have been linked to low calcium absorption, thereby negatively affecting bone health.^19^ This lack of association might stem from potential differences in analysis techniques and population characteristics. Similarly, current smoking habits, while being identified as a significant factor that negatively impacts bone health in multiple studies, had no significant association with bone health in this study.^2^

Numerous studies and organizations have consistently demonstrated an elevated risk of both traumatic and non-traumatic fractures in individuals with low bone mineral density (BMD). The National Osteoporosis Risk Assessment (NORA) study, reported that individuals with osteoporosis are four times more likely to experience fractures compared to those with normal BMD. Similarly, individuals with osteopenia have a 1.8 times higher fracture rate.^21^ These findings underscore the critical importance of conducting risk assessments and identifying factors that contribute to low BMD. Timely interventions are essential to prevent potentially devastating consequences in the future. This is particularly significant in countries where access to good nutrition and health education may be limited, as such interventions can play a pivotal role in addressing the impact of osteoporosis.

The generalizability of the study is limited by the exclusion of non-ambulatory women and women undergoing hormone replacement therapy, whose health outcomes may differ. Additionally, participants were recruited through information disseminated via various mass communication mediums (radio, social media, etc) that might have excluded women who were unable to receive the information. Only women who were able and willing to travel to the hospital were included, potentially excluding those with limited mobility, resources, or time, emphasizing the need for a more diverse sample in future research. Further confirmation of risk factors requires a case-control study.

## CONCLUSIONS

The study reports a lower prevalence of osteoporosis than expected in postmenopausal women. There was a significant inverse relationship between osteoporosis and BMI, the similar findings were observed with BMD. There was also a significant association observed between age and gravida with osteoporosis in postmenopausal women. It emphasizes the significance of good nutrition, health education, and robust health assessment to support bone health and prevent osteoporosis-related complications. Healthcare professionals should consider age, BMI, and menopausal history during risk assessments to identify individuals at higher risk for osteoporosis.
